# CircHIPK3′s dual role in promoting angiogenesis and inhibiting apoptosis through FASN mRNA stabilization in gallbladder cancer

**DOI:** 10.1016/j.clinsp.2025.100697

**Published:** 2025-06-03

**Authors:** ZhenWei Li, HaiJiu Wang, Cheng Wang, ZhiXin Wang, Zhi Xie, JinMing Liu

**Affiliations:** a Department of Hepatopancreatobiliary Surgery, Qinghai University Affiliated Hospital, Xining City, Qinghai Province, China; b Qinghai Provincial Key Laboratory of Hydatid Disease Research, Xining City, Qinghai Province, China

**Keywords:** CircHIPK3, ALYREF, FASN, Ubiquitination, Gallbladder cancer

## Abstract

**Objective:**

To investigate the mechanism by which circHIPK3 enhances FASN stability by inhibiting the ubiquitinated degradation of ALYREF on Gallbladder Cancer (GBC).

**Methods:**

RT-qPCR was performed to detect circHIPK3 in GBC tissues and to analyze the relationship between circHIPK3 and clinicopathological characteristics of GBC patients. CCK-8 and colony formation assays were performed to study the proliferation of GBC cells, flow cytometry to detect apoptosis, tube formation assay to test angiogenesis, and Transwell assay to study migration and invasion. A nude mice implantation assay was conducted. CircHIPK3, FASN and SKP2 were assessed using RT-qPCR and western blot. Ubiquitination assay was performed to detect ALYREF ubiquitination, and RNA pull-down and RIP to investigate the binding relationship between circHIPK3, ALYREF and FASN.

**Results:**

CircHIPK3 was upregulated in GBC tissues, with elevated expression correlating with TNM stages and lymph node metastasis. CircHIPK3 knockdown suppressed GBC progression by inhibiting proliferation, angiogenesis, migratory and invasive capacity while promoting apoptosis *in vitro*, paralleled by reduced tumor growth *in vivo*. CircHIPK3 stabilized ALYREF via inhibiting ubiquitination-mediated degradation. ALYREF knockdown phenocopied the tumor-suppressive effects of circHIPK3 depletion, whereas ALYREF overexpression rescued circHIPK3 knockdown-induced suppression of malignant phenotypes. CircHIPK3 stabilizes FASN mRNA through ALYREF. Overexpressing FASN counteracted the actions of circHIPK3 downregulation on cellular angiogenesis and apoptosis.

**Conclusion:**

CircHIPK3 enhances FASN stability by inhibiting ALYREF ubiquitination degradation, promoting angiogenesis, and inhibiting apoptosis in GBC. This study positions circHIPK3 as a promising prognostic biomarker and therapeutic target in GBC, with ALYREF and FASN emerging as critical mechanistic nodes within this oncogenic pathway.

## Highlights

• Nude mice implantation experiment.
• RNA FISH-immunofluorescence microscopy.
• Cycloheximide (CHX) and protease inhibitor MG132 treatment assay.

## Introduction

Gallbladder Cancer (GBC) ranks among the most prevalent malignancies of the biliary system, yet constitutes a comparatively uncommon malignancy relative to other systemic tumors.^1^ GBC demonstrates significant geographic and ethnic variability in global incidence patterns, with a pronounced female predominance. Despite its relatively low overall prevalence, the disease is characterized by clinically insidious presentation, aggressive tumor biology, and poor prognosis.^2^^,^^3^ Radical resection may be the most effective strategy for GBC, but it is only applicable to a small proportion of patients with early diagnosed GBC.^1^ More than half of the new GBC cases are detected after a laparoscopic cholecystectomy performed for an assumed benign ailment, and the majority of patients lack specific clinical manifestations in the early stages of GBC or present with jaundice, abdominal pain, and other signs indistinguishable from gallstones or cholecystitis. GBC has a strong invasive and metastatic ability, easily metastasizing to organs such as the liver or adjacent colon, which are extremely rich in blood supply, posing a great challenge to both surgical and non-surgical treatments.^4^^,^^5^ Therefore, further elucidation of the biological characteristics of GBC in the process of development and metastasis, and providing new ideas for its early diagnosis and treatment are of great significance and have become the key issues in the field of biliary malignant tumors that need to be solved urgently.

CircRNAs are a subclass of non-coding RNAs that arise from reverse splicing and are characterized by a covalent closed-loop structure.^6^ Thousands of circRNAs have been identified and found to have tissue-specific expression patterns.^7^ Investigations have demonstrated the vital biological roles of circRNAs, which are implicated in the regulation of diseases. CircRNAs primarily function by inhibiting miRNAs and interacting with RNA Binding Proteins (RBPs) to control gene transcription.^8^ Given their stable nature and abnormal expression in pathological conditions, circRNAs are highly potential biomarkers for tumor diagnosis and prognosis.^9^

Derived from exon 2 of the HIPK3 gene on chromosome 11p13, circRNA Homeodomain-Interacting Protein Kinase 3 (circHIPK3) consists of extended introns of HIPK3, each containing reverse Alu repeat sequences.^10^ circHIPK3 plays an essential role in cancer onset, progression, and metastasis.^11^ Inhibition of mRNA translation by circHIPK3 is possible at the post-transcriptional level by regulating miRNA expression.^12^ In a GBC-related study, circHIPK3 expression has been measured to up-regulate in GBC cells and promote GBC cell growth through adsorption of miR-124.^13^ Recently, more and more studies have begun to focus on the interaction of circRNAs with RBPs and their effects on tumors.^14^^,^^15^ However, there is little proof that circHIPK3 directly binds or regulates RBP.

Therefore, this study probed the relationship between circHIPK3 and ALYREF/FASN, as well as their interactions in GBC, hoping to provide a new theoretical basis for the early diagnosis and treatment of GBC.

## Materials and methods

### Case specimens

Samples of 46 GBC patients from Qinghai University Affiliated Hospital between June 2019 and October 2021 were collected. A clinical diagnosis was made for all patients, and preoperative radiotherapy was not given to any of them. The surgically excised tissues (cancerous tissue and paracancerous tissue more than 5 cm from the margins of the cancerous tissues) were frozen in liquid nitrogen. This study was approved by the Ethics Committee of Qinghai University Affiliated Hospital (n° 20181125QH23), and the subjects signed an informed consent form. The clinicopathologic characteristics of all included patients are shown in Table 1.

**Table 1:** Relationship of circHIPK3 expression with clinicopathological characteristics in 46 cases GBC patients.

**Clinicopathological characteristics**	**Patients (*n* = 46)**	**circHIPK3**		**p**
		**Lowe (*n* = 23)**	**High (*n* = 23)**	
Age				
≤ 60	29	13	16	0.360
> 60	17	10	7	
Gender				
Male	16	9	7	0.536
Female	30	14	16	
Tumor size				
≤ 5 cm	18	8	10	0.546
> 5 cm	28	15	13	
Lymph node metastasis				
N_0_	21	14	7	0.038
N_1_~N_3_	25	9	16	
TNM stage				
I‒II	20	6	14	0.017
III‒IV	26	17	9	
Histological grade				
well and moderately	23	8	9	0.760
Poorly and others	23	15	14	

### Cell culture

Human Intrahepatic Biliary Epithelial Cells (HIBEpiCs), Human Umbilical Vein Endothelial Cells (HUVECs), and human gallbladder carcinoma cell lines NOZ and GBC-SD were obtained from the American Type Culture Collection (ATCC). DMEM (Gibco, USA) containing 10 % FBS (Gibco) and 1 % penicillin/streptomycin 100 U/m L (Gibco) was used for cell culture at 37 °C, 5 % CO_2_ and saturated humidity. All experiments were conducted on logarithmic growth phase cells.

### Cell transfection

Two shRNAs targeting circHIPK3 (sh-circHIPK3#1, sh-circHIPK3#2), two shRNAs targeting ALYREF (sh-ALYREF#1 and sh-ALYREF#2), and non-specific shRNA were obtained (GenePharma, Shanghai, China). Overexpression plasmid vectors (pcDNA3.1-ALYREF and pcDNA3.1-FASN) were constructed by inserting the coding sequence of ALYREF and FASN into pcDNA3.1(+) vector (GenePharma), with empty vector (pcDNA3.1) as negative control (GenePharma). NOZ cells were spread over 12-well plates 24 h before transfection and cultured to 60 % confluence in 1.5 mL of antibiotics-free complete culture medium. Transient transfection was conducted under the mediation of liposomal lipofectamine 2000 (Invitrogen, USA). The culture medium was changed 6 h later, and cells were cultured for 48 h. Transfection efficiency was verified using RT-qPCR or Western blot.

### CCK-8

In the logarithmic phase, trypsin digestion was performed on NOZ cells, and cells were suspended in a complete medium. The 96-well plates were covered with 200 μL complete medium, in which NOZ cells were cultured at 4 × 10^3^/well. After 0 h, 24 h, 48 h, and 72 h, CCK-8 reagent (Beyotime, Shanghai, China) was added at 10 μL/well, and the absorbance at 450 nm after 2 h was read.

### Colony formation experiment

After digestion and density adjustment, NOZ cells (300 cells/mL) were put into a 6 cm diameter Petri dish and distributed evenly by shaking. Following the 10-day culture, the dish was rinsed with PBS and cells were fixed with paraformaldehyde, stained with crystalline violet for 2 min, and observed under the inverted microscope (Olympus, Japan). Colonies formed in each dish were calculated.

### Flow cytometry

After digestion, cells were centrifuged and resuspended in PBS to 1 × 10^6^/mL. About 200 μL of the cell suspension underwent two washes with 1 mL of pre-cooled PBS and was centrifuged. The cells were then resuspended in 100 μL of binding buffer, and 2 μL of Annexin-V-FITC (20 μg/mL) was added, allowing it to react on ice for 15 min, away from light. Next, cells were mixed with 300 μL PBS, and apoptotic analysis was done on the BD FACSVerse flow cytometer (BD Biosciences, USA) within 30 min after the addition of 1 μL PI (50 μg/mL).

### Transwell assay

Cells were resuspended with serum-free culture medium and put in Matrigel-lined Transwell (Corning, USA). About 5 × 10^4^ cells were inoculated at 500 μL/well on the top chamber (filled with 10 % FBS) for 30 h. The cells invading to the bottom chamber were fixed with paraformaldehyde and stained with crystal violet, and the number of cells in each field of view was counted under the microscope (Olympus). The migration assay was performed without Matrigel coating, and the rest of the procedure was the same as that of invasion.

### Tube formation assay

Cells were starved in serum-free medium for 24 h, which was then replaced with 10 % FBS medium. The supernatant was centrifuged at 1000 r/min for 10 min and then filtered through a 0.22 μm filter to form a conditioned medium, which was stored at 4 °C. The conditioned medium was mixed with Matrigel (Corning) at 1:1, and 40 μL medium was added to the 96-well plate. On the next day, HUVECs were digested and resuspended to 1 × 10^5^ cells/mL, and added to the 96-well plate at 200 μL/well, after which the cells were cultured in the incubator, photographed and counted after the tubes were visualized by microscope (Olympus).

### Nude mice implantation experiment

Ten nude mice (female, 4-weeks old) were selected from HFKBio (Beijing, China) and kept in a strictly sterilized laminar-flow animal room (SPF grade), with the indoor temperature maintained at 25 °C and the humidity at 60 %‒70 %. Cell suspension (200 μL) was aspirated and injected into the subcutaneous right dorsal side of nude mice. Starting from the date of cell inoculation, the longest diameter (length) and the shortest diameter (width) of the tumors were measured 7d later using a ruler sterilized by ultraviolet light, and then every 7d thereafter. Tumor volume was calculated as 0.5 × length × width^2^, and the tumor growth curve was plotted. After 21 days, the nude mice were euthanized using CO_2_, the subcutaneous tumors were excised whole and photographed, and the tumors were weighed. All procedures were approved by the Ethics Committee of Qinghai University Affiliated Hospital (n° 20190618QH10) and followed the ARRIVE guidelines.

### Immunohistochemistry (IHC)

Tumor tissues were fixed in 4 % paraformaldehyde, sequentially dehydrated, embedded in paraffin, and sectioned at 4 μm. The sections were routinely deparaffinized and hydrated, endogenous peroxidase was eliminated by H_2_O_2_, and antigen was retrieved by microwave. Sections were blocked with 10 % normal goat serum for 30 min, and primary antibody Ki-67 (1:100, Abcam, USA) or CD31 (1:100, Abcam) was incubated at room temperature for 60 min, followed by secondary antibody (Proteintech, Wuhan, China) for 30 min. Sections were then stained with hematoxylin after DAB color development and sealed with neutral resin. At magnification of 400 times, 5 fields of view were randomly selected for taking images which were analyzed using Image J to calculate the number of positive cells in each field of view.

### RT-qPCR

After the total RNA of tissues and cells was extracted by Trizol kit (Invitrogen, USA), it was dissolved in double-distilled water treated with DEPC, and then the concentration and purity were determined by DU-800 Nucleated Protein Analyzer (Beckman, USA). PCR primers (Table 2) were synthesized by GenePharma. RNA was reverse transcribed into cDNA using RNA Reverse Transcription Kit (Takara, Japan), followed by PCR amplification with the SYBR Green PCR Master Mix (Thermo Fisher Scientific) and electrophoresis on agarose gels. Data were analyzed by the 2^-ΔΔCt^ method.

**Table 2:** Primer sequences.

**Genes**	**Sequences (5′‒3′)**
CircHIPK3	Forward: TATGTTGGTGGATCCTGTTCGGCA
	Reverse: TGGTGGGTAGACCAAGACTTGTGA
HIPK3	Forward: TCACAAGTCTTGGTCTACCCA
	Reverse: CACATAGGTCCGTGGATAGTTTC
ALYREF	Forward: CTCCCAGGAACTCTTTGCTGAA
	Reverse: CTGCCTTCCGCTCAAAGTG
FASN	Forward: CCCCTGATGAAGAAGGATCA
	Reverse: ACTCCACAGGTGGGAACAAG
GAPDH	Forward: GAGTCAACGGATTTGGTCGT
	Reverse: TGTGGTCATGAGTCCTTCCA

### Western blot assay

Total proteins were extracted using the RIPA kit (Beyotime). According to the results of protein quantification using the BCA Protein Concentration Assay Kit (Beyotime), the samples were processed by adding 10 % SDS-PAGE for 2 h and 5 % skimmed milk powder for 2 h. The primary antibodies were: rabbit anti-ALYREF (1:1000, ab202894, Abcam), rabbit anti-FASN (1:1000, A19050, Abclonal, China), rabbit anti-Bax (1:1000, ab32503, Abcam), rabbit anti-Bcl-2 (1:1000, ab196495, Abcam), mouse anti-VEGFA (1 µg/mL, ab155944, Abcam), mouse anti-ubiquitin (1:500, Cat#3936, Cell Signaling Technology, USA), and GAPDH (1:10,000, 60,004–1-Ig, Proteintech). After overnight incubation at 4 °C, proteins were combined with HRP-labeled goat anti-rabbit lgG secondary antibody (1:2000, Cat#31,460, Invitrogen) or goat anti-mouse lgG (1:1000, Cat#31,430, Invitrogen) for 1 h and developed. Gray values were analyzed by Image Lab.

### RNA FISH-immunofluorescence microscopy

FISH Kit was used (GenePharma) in the assay. Cells were fixed in 4 % paraformaldehyde for 10 min and added with 1 mL PBS containing 0.5 % Triton X-100 at 4° for 5 min. Prehybridization solution (200 μL) was added to each well for 30 min, and Cy3-circHIPK3 probe (2.5 μL, 20 μmoL/L, GenePharma) was preheated in the hybridization solution at 37 °C under light-avoidance conditions. Then, 100 μL probe hybridization solution containing the probe was incubated avoid light, and hybridized at 37 °C overnight. The excess liquid was removed, and cells were restained by adding a DAPI staining solution for 15 min. Cells were blocked, observed, and photographed under the fluorescence microscope.

### Subcellular isolation experiments

Nuclei and cytoplasmic components of cells were isolated using PARIS kits (Life Technologies, USA). Cells (2 × 10^6^) were washed twice with ice PBS and supplemented with 450 μL of cell isolation buffer. Subsequently, the compounds were incubated on ice for 5 min followed by centrifugation at 4 °C for 5 min. Finally, RNA was collected from the precipitated nuclei and cytoplasm in the supernatant, respectively. U6 and GAPDH were internal references in the nucleus and cytoplasm, respectively.

### RNase R treatment assay

RNA was isolated and treated with RNase *R* (3 U/μg RNA, Geneseed, Guangzhou, China) for half an hour at 37 °C. Subsequently, the RNA underwent purification through the RNeasy MinElute Cleaning Kit (Qiagen, Germany). Ultimately, circHIPK3 and linear HIPK3 were examined using RT-qPCR.

### Actinomycin D assay

Cells were inoculated in 6-well plates at 5 × 10^5^ cells/well, and actinomycin D (Sigma, USA) was added to each well at 2 μg/mL at 0 h, 4 h, 8 h, and 12 h. Cells were collected at the indicated time points. Changes in RNA expression over time were analyzed using RT-qPCR.

### RNA pull-down

The Pierce Magnetic RNAProtein pull-down Kit (Thermo Fisher Scientific, USA) was utilized to conduct RNA pull-down tests. The RNA underwent labeling using a biotin-tagged circHIPK3 Probe (GenePharma) on M-280 Streptavidin Magnetic Beads (Sigma), followed by blending with cell lysate at 4 °C for an hour. Subsequently, the beads were cleansed twice using lysis buffer, three times with low-salt buffer, and once with high-salt buffer. Subsequently, the RNA-binding protein complexes underwent western blot analysis to detect ALYREF.

### Cycloheximide (CHX) and protease inhibitor MG132 treatment assay

Cells transfected with sh-NC, sh-circHIPK3#1, or sh-circHIPK3#2 were inoculated at 1 × 10^6^/well in 6-well plates, and CHX (50 µg/mL, Beyotime) was added. At 0 h, 2 h, and 4 h, a western blot was conducted for ALYREF expression. Cells were collected at 0 h, 2 h, and 4 h intervals and analyzed by western blot for ALYREF expression. Or MG132 (5 µM, Beyotime) was added, and cells were collected 12 h later for analysis of ALYREF.^16^

### Ubiquitination assays/Co-IP

Cells transfected with sh-NC, sh-circHIPK3#1, or sh-circHIPK3#2 were lysed with RIPA lysis buffer (Beyotime) supplemented with protease inhibitors and phosphatase inhibitors for 1 h at 4 °C. Cells were centrifuged at 15,000 rpm for 15 min, followed by the collection and subsequent incubation of the supernatant protein lysate with designated antibodies for 2 h at 4 °C, employing homologous IgG as the benchmark. Subsequently, magnetic beads (Magnetic Protein A/G beads) (Beyotime) were added to the mixture and rotated for 12 h at 4 °C. Finally, SDS buffer was added and boiled at 100 °C for 8‒10 min, the magnetic beads were adsorbed, and the supernatant was taken for subsequent Western Blot for ubiquitination of ALYREF protein.^17^

### RIP

The EZ-Magna RIP kit (Millipore, USA) was utilized to conduct RIP assays. The cells underwent lysis using RIP lysis buffer, followed by a 24-hour incubation at 4 °C with RIP buffer, which included magnetic beads conjugated to either ALYREF antibody or IgG (5 μg, Abcam). Subsequently, the immunoprecipitated RNA from the beads was isolated for RT-qPCR analysis.

### Statistical analysis

All data were processed using GraphPad Prism 9.0 (GraphPad Software, USA), and expressed in the form of mean ± standard deviation. The relationship between circHIPK3 expression and clinicopathological characteristics of GBC patients was determined by the Chi-Square test. Comparisons between two groups were *t*-test, and comparisons between multiple groups were performed by One-Way ANOVA and Tukey's multiple comparisons test; *p* < 0.05 indicated that the differences were statistically significant.

## Results

### circHIPK3 is upregulated in GBC

circHIPK3 (circRNA ID: hsa_circ_0000284) consists of a larger second exon (1099 nt) of the HIPK3 gene, which is flanked by long introns on each side.^18^ To characterize its tumor-associated expression, the authors performed pan-cancer analysis using the MiOncoCirc database, which revealed elevated circHIPK3 levels in multiple malignancies, particularly Adrenocortical Carcinoma (ACC), Esophageal Carcinoma (ESCA), and Pancreatic Adenocarcinoma (PAAD) (Supplementary Fig. 1A). Given the scarcity of gallbladder cancer-specific circRNA datasets, the authors analyzed the most relevant publicly available resource ‒ the GSE148561 dataset comprising six paired distal cholangiocarcinoma samples. Using the circMine platform, the authors observed no statistically significant differential expression of circHIPK3 between tumor and adjacent normal tissues in this biliary tract malignancy cohort (Supplementary Fig. 1B). Notably, Wang et al.^19^ previously demonstrated that circHIPK3 acts as a competitive endogenous RNA to drive cholangiocarcinoma progression. Furthermore, prior evidence supports its role as a potential therapeutic target and diagnostic marker in GBC.^13^ These findings, combined with its consistent overexpression across diverse malignancies, established a compelling rationale for prioritizing its mechanistic exploration in GBC.

**Fig. 1:**
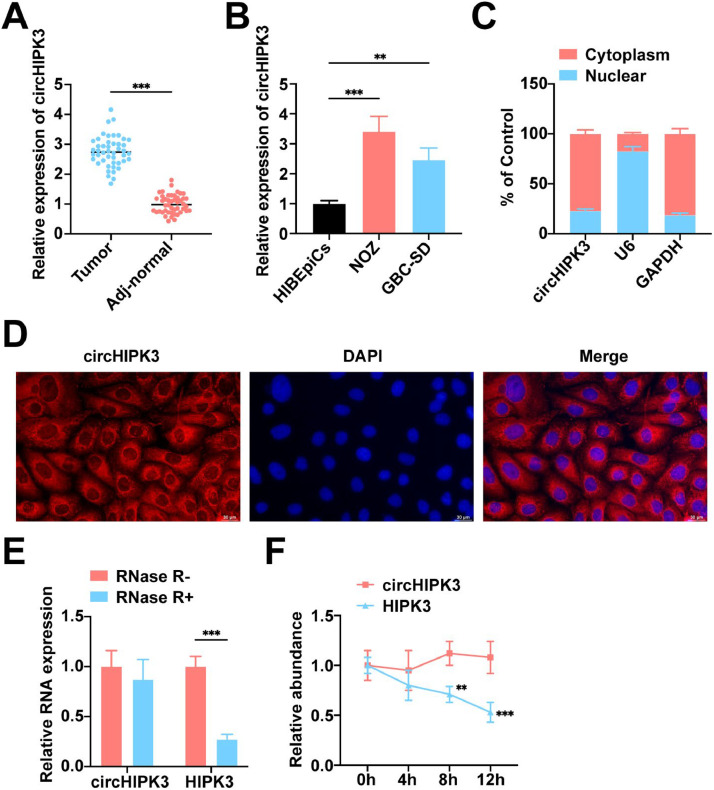
**CircHIPK3 is upregulated in GBC.** (A) RT-qPCR to detect circHIPK3 in GBC tissues and paracancerous tissues, *n* = 46. (B) RT-qPCR to detect the expression level of circHIPK3 in HIBEpiCs and human gallbladder carcinoma cell lines. (C) circHIPK3 expression in nucleus and cytoplasm in NOZ cells. (D) FISH analysis of circHIPK3 in NOZ cells. (E) RNase R assay to verify the circular structure of circHIPK3. (F) Actinomycin D assay to detect the stability of circHIPK3 mRNA. All experiments were repeated at least three times. * *p* < 0.05, ** *p* < 0.01, and *** *p* < 0.001.

The authors collected 46 cases of GBC tissues and paracarcinoma tissues for testing and found that circHIPK3 expression was significantly upregulated in GBC tissues compared with paracarcinoma tissues (Fig. 1A). Table 1 displays the clinicopathological information for 46 GBC patients, categorizing them into the circHIPK3 low-expression and high-expression groups according to the median circHIPK3 levels. The statistical evaluation revealed a strong link between elevated circHIPK3 expression in GBC patients and lymph node metastasis and TNM stages (III‒IV), though this was not the case with other variables. RT-qPCR results indicated that circHIPK3 levels were higher in NOZ and GBC-SD cell lines compared to HIBEpiCs cells, with the most significant increase observed in NOZ cells (Fig. 1B), leading to the selection of this cell line for further experiments. Nucleoplasmic separation as well as FISH assay showed that circHIPK3 was mainly localized in the cytoplasm of NOZ cells (Fig. 1C‒D). Resistance to RNase R indicated that circHIPK3 has a closed-loop structure (Fig. 1E). Actinomycin D treatment revealed that circHIPK3 remained stable compared to linear HIPK3 (Fig. 1F).

### circHIPK3 downregulation inhibits GBC angiogenesis and promotes apoptosis

**Fig. 2:**
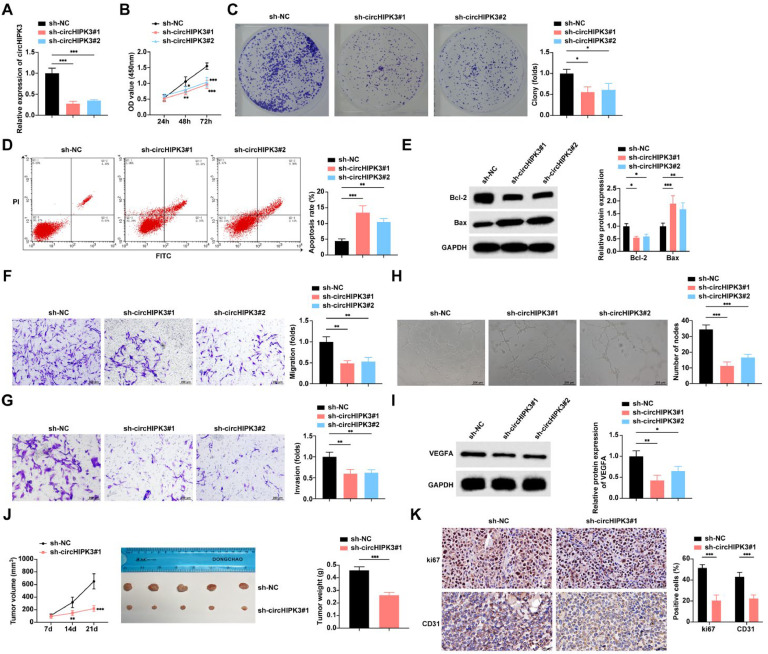
**Down-regulation of circHIPK3 inhibits GBC angiogenesis and promotes apoptosis.** (A) RT-qPCR to detect the expression level of circHIPK3 in NOZ cells. (B) CCK-8 assay to study the proliferation level of NOZ cells. (C) Colony formation assay to study the rate of clone formation in NOZ cells. (D) Flow cytometry to study the rate of apoptosis in NOZ cells; (E) Western blot to detect the protein expression of Bax and Bcl-2 in NOZ cells. (F‒G) Transwell assay to study the migration and invasion levels of NOZ cells. (H) Tube formation assay to assess the angiogenic capacity of NOZ cells. (I) Western blot to detect the protein expression of VEGFA in HUVECs. (J) Nude mouse implantation assay to detect the tumor growth volume and weight of NOZ cells, *n* = 5. (K) IHC to assess the expression levels of Ki67 and CD31 in transplanted tumor tissues in nude mice, *n* = 5. All experiments were repeated at least three times. * *p* < 0.05, ** *p* < 0.01, and *** *p* < 0.001.

Both sh-circHIPK3#1 and sh-circHIPK3#2 successfully knocked down circHIPK3 in the cells (Fig. 2A). CCK-8 and colony formation assays found that cell proliferation and the rate of colony formation were decreased after circHIPK3 downregulation (Fig. 2B‒C). Flow cytometry and Western blot studies reported that circHIPK3 downregulation increased the apoptosis rate and Bax expression in tumor cells and suppressed Bcl-2 expression (Fig. 2D‒E). Cell migration and invasion were hindered after transfection with sh-circHIPK3#1 and sh-circHIPK3#2, as shown by the Transwell assay (Fig. 2F‒G). The effect of down-regulation of circHIPK3 on angiogenic ability was further detected by Matrigel *in vitro* tube formation assay using HUVECs. circHIPK3 downregulation inhibited the formation of tube-like structures (Fig. 2H). Western blot assay further verified that circHIPK3 downregulation inhibited VEGFA expression, an angiogenesis-related factor, in HUVECs (Fig. 2I).

A mouse xenograft tumor model was developed to study the influence of circHIPK3 on GBC *in vivo*. The results confirmed that tumor growth was significantly inhibited after knocking down circHIPK3 expression (Fig. 2J). IHC staining of the tumor tissues taken afterward revealed that Ki67 and CD31 proteins were significantly suppressed when circHIPK3 was downregulated (Fig. 2K‒L).

### circHIPK3 interacts with ALYREF and inhibits its ubiquitination and degradation

To dig into the molecular mechanism, the authors identified ALYREF as a top ranked RBP interacting with circHIPK3 using the ENCORI platform, selected from 10 candidate RBPs based on the highest ClipSiteNum score (Supplementary Table 1). This prediction aligns with prior experimental evidence highlighting ALYREF’s critical role in circRNA-protein interactions, particularly in regulating RNA stability and nuclear export mechanisms.^20^^,^^21^ To further validate its clinical relevance, the authors analyzed cholangiocarcinoma data from the GEPIA2 database, which revealed significant ALYREF upregulation in tumor tissues compared to normal controls (Supplementary Fig. 2A), consistent with its established oncogenic role across multiple cancer types. Survival analysis showed a non-significant trend toward poorer prognosis in high ALYREF-expressing patients (Supplementary Fig. 2B).

**Fig. 3:**
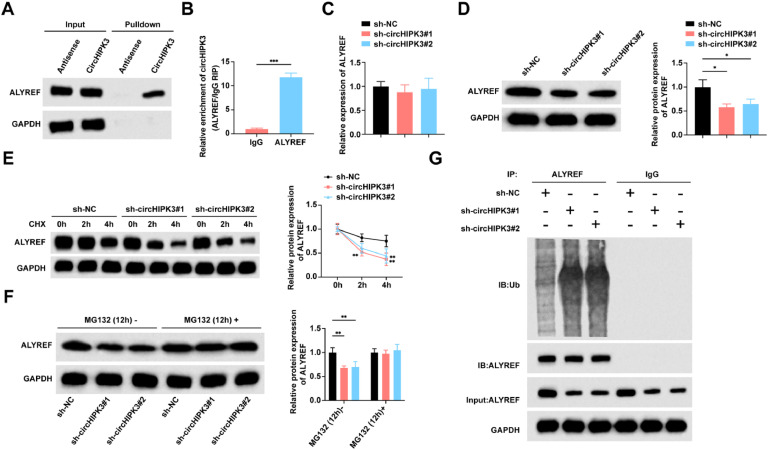
**CircHIPK3 interacts with ALYREF to inhibit its ubiquitination and degradation.** (A) RNA-pull down and Western blot assay confirmed the interaction between ALYREF and circHIPK3. (B) RIP experiments to analyze the enrichment of circHIPK3. (C) RT-qPCR to detect the mRNA expression level of ALYREF in transfected NOZ cells. (D) Western blot to detect the protein expression level of ALYREF in transfected NOZ cells. (E) Western blot to detect the protein expression level of ALYREF in CHX-treated NOZ cells. (F) Western blot to detect the protein expression level of ALYREF in MG132-treated NOZ cells. (G) Immunoprecipitation using anti-ALYREF antibody in transfected NOZ cells, followed by ubiquitination level of ALYREF by Western blot. All experiments were repeated at least three times. * *p* < 0.05, ** *p* < 0.01, and *** *p* < 0.001.

Using a biotin-labeled circHIPK3 probe, the authors specifically extracted circHIPK3 and found ALYREF in the resulting precipitates (Fig. 3A). RIP assay showed that ALYREF protein immunoprecipitates contained circHIPK3 (Fig. 3B). In GBC cells with low expression of circHIPK3, RT-qPCR and Western blot revealed that ALYREF protein was downregulated in cells transfected with sh-circHIPK3#1 and sh-circHIPK3#2, but ALYREF mRNA levels were not significantly changed (Fig. 3C‒D), suggesting that circHIPK3 regulates ALYREF expression at the post-transcriptional level. Subsequently, treatment of cells with a protein synthesis inhibitor (CHX) revealed that circHIPK3 knockdown significantly reduced the half-life of ALYREF protein in cells (Fig. 3E). In contrast, when the proteasome activity of the cells was inhibited with MG132, it was observed that the down-regulation of ALYREF levels originally induced by circHIPK3 knockdown was blocked (Fig. 3F). Based on the fact that the ubiquitin-proteasome pathway is responsible for the degradation of many proteins, the authors performed Co-IP experiments to measure the ubiquitination level of ALYREF when circHIPK3 was knocked down in NOZ cells, and the experiments revealed that the ubiquitination level of ALYREF was markedly elevated when circHIPK3 was knocked down in NOZ cells. circHIPK3 knockdown could destabilize the ALYREF protein by promoting ubiquitination (Fig. 3G).

### ALYREF downregulation inhibits GBC angiogenesis and promotes apoptosis

**Fig. 4:**
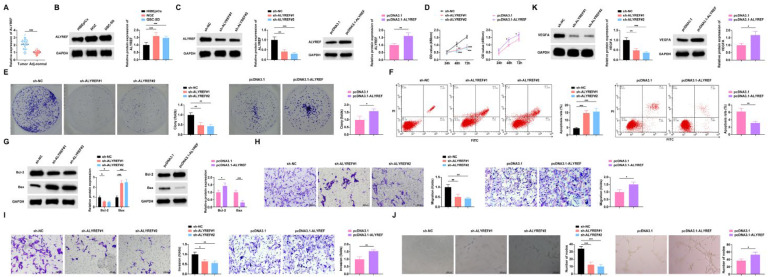
**ALYREF downregulation inhibits GBC angiogenesis and promotes apoptosis.** (A) RT-qPCR to detect the mRNA expression level of ALYREF in GBC tissues and paracancerous tissues, *n* = 46. (B) Western blot to detect the protein expression level of ALYREF in HIBEpiCs and human GBC cell lines. (C) Western blot to detect the protein expression level of ALYREF in NOZ cells. (D) CCK-8 assay to study the proliferation level of NOZ cells. (E) Colony formation assay to study the colony formation rate of NOZ cells. (F) Flow cytometry to study the apoptosis rate of NOZ cells. (G) Western blot to detect the protein expression of Bax and Bcl-2 in NOZ cells. (H‒I) Transwell assay to study the migration and invasion level of NOZ cells. (J) Tube formation assay to assess the angiogenic ability of NOZ cells. (K) Western blot to detect the protein expression of VEGFA in HUVECs. All experiments were repeated at least three times. * *p* < 0.05, ** *p* < 0.01, and *** *p* < 0.001.

RT-qPCR analysis demonstrated significant upregulation of ALYREF expression in Gallbladder Cancer (GBC) tissues relative to adjacent normal tissues (Fig. 4A), with corresponding elevation observed in NOZ and GBC-SD cell lines compared to HIBEpiCs (Fig. 4B). The effect of ALYREF on the malignant phenotype of GBC cells was investigated through ALYREF knockdown (sh-ALYREF#1/#2) and overexpression (pcDNA3.1-ALYREF) in NOZ cells, with Western blot confirmed successful knockdown and overexpression (Fig. 4C). Functional characterization revealed that ALYREF depletion suppressed cell proliferation and colony formation, while its ectopic expression enhanced these malignant properties (Fig. 4D‒E). Flow cytometry and Western blot demonstrated that ALYREF silencing increased apoptosis rates accompanied by elevated Bax and reduced Bcl-2 protein levels, whereas ALYREF overexpression produced opposing effects on cell apoptosis (Fig. 4F‒G). Migration and invasion capacities were markedly attenuated by ALYREF knockdown but augmented by its overexpression (Fig. 4H‒I). Angiogenesis assays showed compromised tube-forming ability in ALYREF-deficient cells, while ALYREF overexpression promoted angiogenic potential (Fig. 4J‒K).

### ALYREF mediates the regulation of circHIPK3 on the malignant phenotype of GBC cells

**Fig. 5:**
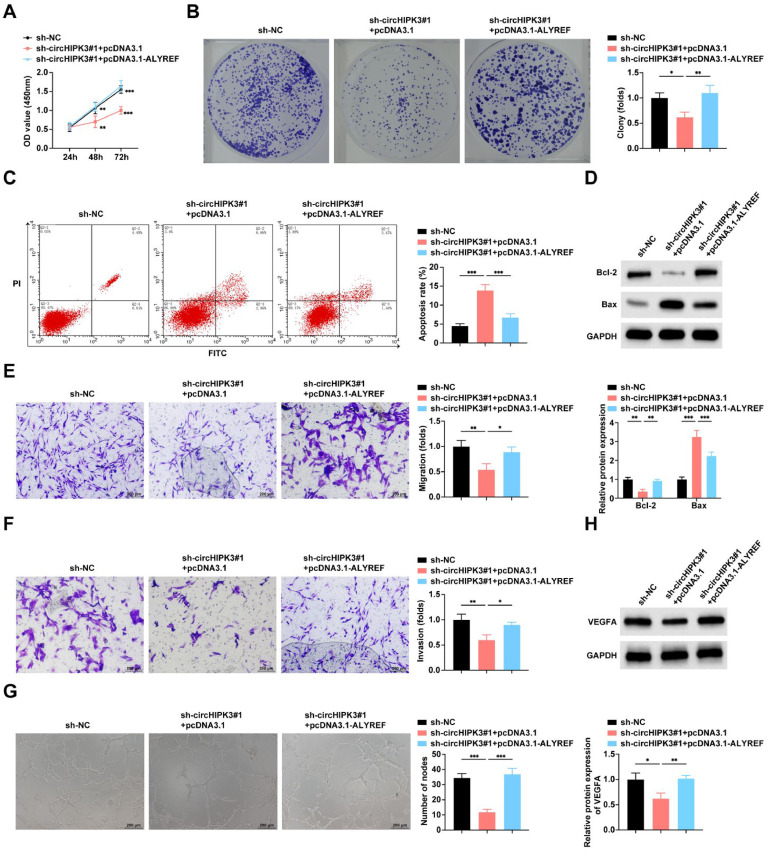
**ALYREF mediates the regulation of circHIPK3 on the malignant phenotype of GBC cells.** (A) CCK-8 assay to study the proliferation level of NOZ cells; (B) Colony formation assay to study the colony formation rate of NOZ cells. (C) Flow cytometry to study the apoptosis rate of NOZ cells. (D) Western blot to detect the protein expression of Bax and Bcl-2 in NOZ cells. (E‒F) Transwell assay to study the migration and invasion level of NOZ cells. (G) Tube formation assay to assess the angiogenic ability of NOZ cells. (H) Western blot to detect the protein expression of VEGFA in HUVECs. All experiments were repeated at least three times. * *p* < 0.05, ** *p* < 0.01, and *** *p* < 0.001.

To investigate the mediating role of ALYREF on circHIPK3 regulation of the malignant phenotype of GBC cells, the authors co-transfected sh-circHIPK3#1 and pcDNA3.1-ALYREF into NOZ cells for rescue experiments. Functionally, ALYREF overexpression rescued the proliferation and colony formation deficits induced by circHIPK3 knockdown in NOZ cells (Fig. 5A‒B). This compensatory effect extended to apoptosis regulation, where ALYREF reconstitution reversed the pro-apoptotic effects of circHIPK3 silencing, as evidenced by decreased apoptotic rates (Fig. 5C) and corresponding modulation of apoptosis markers (reduced Bax and elevated Bcl-2 protein levels) (Fig. 5D). The impaired migratory and invasive capacities caused by circHIPK3 knockdown were similarly restored upon ALYREF over-expression (Fig. 5E‒F). Angiogenic suppression mediated by circHIPK3 depletion was likewise negated by ALYREF reintroduction (Fig. 5G‒H). Collectively, these rescue experiments demonstrate that circHIPK3 regulates malignant behaviors in GBC cells through ALYREF-dependent mechanisms.

### circHIPK3 stabilizes FASN mRNA through interaction with ALYREF

**Fig. 6:**
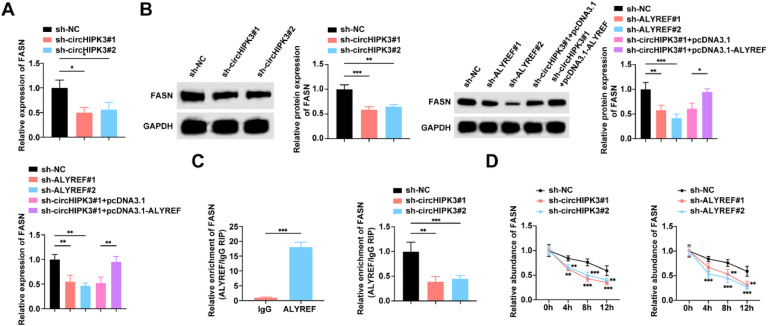
**CircHIPK3 stabilizes FASN mRNA through interaction with ALYREF.** (A) RT-qPCR to detect the mRNA expression level of FASN in NOZ cells. (B) Western blot to detect the protein expression of FASN in NOZ cells. (C) RIP experiments to detect the enrichment of FASN. (D) RT-qPCR to detect the stability of FASN mRNA in NOZ cells after actinomycin D treatment. All experiments were repeated at least three times. * *p* < 0.05, ** *p* < 0.01, and *** *p* < 0.001.

The authors next investigated the downstream genes of circHIPK3/ALYREF. circHIPK3 can regulate FASN through a ceRNA mechanism.^22^ Using ENCORI (https://rnasysu.com/encori/index.php), ALYREF and FASN were predicted to have a binding relationship. Both circHIPK3 and ALYREF knockdown decreased FASN gene and protein expression in GBC cells by RT-qPCR assay, whereas overexpression of ALYREF impaired the suppression of FASN gene and protein expression by circHIPK3 knockdown (Fig. 6A‒B). The authors then validated the interaction between ALYREF and FASN, and the RIP results showed that FASN mRNA was enriched in ALYREF immunoprecipitation complexes in NOZ cells, while knockdown of circHIPK3 reduced the binding of ALYREF to FASN mRNA (Fig. 6C). CircHIPK3/ALYREF knockdown reduced FASN mRNA stability by Actinomycin D assay (Fig. 6D).

### circHIPK3 inhibits GBC cell angiogenesis and promotes apoptosis through FASN

**Fig. 7:**
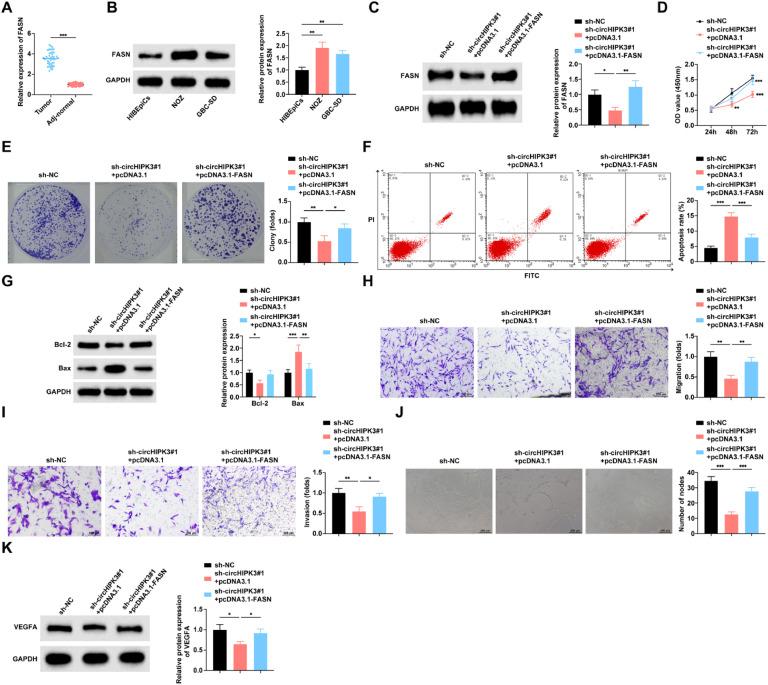
**CircHIPK3 inhibits GBC cell angiogenesis and promotes apoptosis through FASN.** (A) RT-qPCR to detect the mRNA expression level of FASN in GBC tissues and paracancerous tissues, *n* = 46. (B) RT-qPCR to detect the mRNA expression level of FASN in HIBEpiCs and human GBC cell lines. (C) Western blot to detect the protein expression level of FASN in NOZ cells. (D) CCK-8 assay to study the proliferation level of NOZ cells. (E) Colony formation assay to study the colony formation rate of NOZ cells. (F) Flow cytometry to study the apoptosis rate of NOZ cells. (G) Western blot to detect the protein expression of Bax and Bcl-2 in NOZ cells; (H‒I) Transwell assay to study the migration and invasion level of NOZ cells. (J) Tube formation assay to assess the angiogenic ability of NOZ cells. (K) Western blot to detect the protein expression of VEGFA in HUVECs. All experiments were repeated at least three times. * *p* < 0.05, ** *p* < 0.01, and *** *p* < 0.001.

RT-qPCR assay found that FASN expression was up-regulated in GBC tissues compared to paracarcinoma tissues (Fig. 7A). Similarly, FASN expression was significantly elevated in NOZ and GBC-SD cell lines compared to HIBEpiCs cells (Fig. 7B). Based on previous mechanistic studies validating that circHIPK3 stabilizes FASN mRNA by binding to ALYREF, the authors co-transfected sh-circHIPK3#1 and pcDNA3.1-FASN into NOZ cells for rescue experiments to further explore the role played by FASN in circHIPK3 regulation of malignant biological phenotypes in GBC cells. Western blot verified the transfection efficiency (Fig. 7C). The effect of circHIPK3 knockdown on cell proliferation, colony formation (Fig. 7D‒E), apoptosis (Fig. 7F‒G), migration and invasion (Fig. 7H‒I), and angiogenic capacity (Fig. 7J‒K) was weakened by raising FASN levels.

## Discussion

CircHIPK3, previously documented as upregulated in gastric cancer,^23^ hepatocellular carcinoma,^24^ and biliary tract cancer,^25^ is now demonstrated to be significantly elevated in GBC tissues and cell lines through this investigation. The present findings reveal that heightened circHIPK3 expression correlates with advanced TNM staging and lymph node metastasis in GBC patients. While the cohort size remains limited, these clinical associations suggest circHIPK3′s potential diagnostic utility pending validation in larger patient populations. Subsequently a series of *in vitro* functional assays established that circHIPK3 depletion suppresses GBC cell proliferation and angiogenesis, which is a critical process in tumor survival given that hypoxia-driven tumor progression depends on neovascularization for nutrient supply and waste removal.^26^ This mechanistic understanding aligns with the xenograft experiments showing reduced tumor growth and vascularization following circHIPK3 knockdown. The concordance between these results and prior reports^13^ strengthens the evidence positioning circHIPK3 as both a promising diagnostic biomarker and therapeutic target in GBC management.

RBPs crucially mediate circRNA functions, with growing evidence highlighting circRNA-RBP interactions in tumor progression.^27^ Prior studies demonstrate this regulatory axis in cancer biology: circFOXP1 was shown to bind PTBP1, stabilizing PKLR mRNA to enhance the Warburg effect in GBC,^28^ while circ_0021727 recruits EIF4A3 to stabilize GBX2 mRNA, driving angiogenesis in esophageal squamous carcinoma.^29^ The regulation of cells, whether physiological or pathological, involves RBPs, which participate in modification, stabilization, degradation, and translation.^30^ Emerging research highlights RNA modifications as pivotal regulators in carcinogenesis, with 5-methylcytosine (m5C) being mechanistically associated with cancer progression due to its epigenetic regulatory capabilities. This modification dynamically regulates molecular functions including mRNA processing, splicing, nuclear export, translation efficiency, and stability maintenance, operating under the coordinated control of m5C writers, erasers, and readers. Notably, the RBP ALYREF is considered one type of reader protein located in the nucleus that recognizes and binds directly with m5C sites in RNA and facilitates the export of RNA from the nucleus to the cytoplasm.^31^, ^32^, ^33^ ALYREF is expressed abnormally in multiple cancers, and this abnormal expression can lead to malignant characteristics through mechanisms like pre-mRNA processing and mRNA stabilization.^34^ This study identifies ALYREF as a functional partner of circHIPK3 in GBC. Mechanistically, circHIPK3 binds and stabilizes ALYREF by blocking its ubiquitination. Functional assays confirm that ALYREF knockdown suppresses GBC cell malignancy, while its overexpression exacerbates tumorigenic behaviors, mirroring its oncogenic roles in gastric^35^ and hepatocellular cancers.^33^ Crucially, ALYREF reconstitution counteracts the tumor-suppressive effects of circHIPK3 depletion, establishing that ALYREF mediated the pro-carcinogenic effects of circHIPK3 on GBC.

Building on prior evidence that circHIPK3 modulates FASN expression via miRNA sponging to drive esophageal squamous carcinoma progression,^22^ the bioinformatic analyses identified putative ALYREF-FASN binding interactions. Experimental validation confirmed that circHIPK3 regulates FASN mRNA stability and expression through ALYREF-mediated mechanisms. This discovery motivated a focused investigation of FASN as a downstream mediator within the circHIPK3/ALYREF regulatory axis. As the pivotal enzyme driving de novo lipogenesis, FASN enables the heightened lipid biosynthesis that cancer cells critically depend on to fuel their rapid proliferation, a metabolic adaptation absent in normal cellular physiology.^36^ FASN overexpression in malignancies correlates with poor prognosis, reflecting its essential role in maintaining cancer cell survival and proliferation.^37^ Mechanistically, by enabling the building of lipid rafts, FASN consequently allows the localization of oncogenic receptors like HER2, c-Met, and VEGFR-2 in membrane microdomains, thereby enhancing proliferative, metastatic, and angiogenic signaling.^38^ Migita et al. demonstrated an inverse correlation between FASN expression and apoptotic rates in prostate cancer tissues,^39^ while curcumin-mediated FASN inhibition or genetic silencing in breast cancer cells decreased anti-apoptotic Bcl-2 and increased pro-apoptotic Bax levels.^40^ The pro-angiogenic capacity of FASN is evidenced by its correlation with microvessel density in gliomas^41^ and the angiogenic suppression observed in colorectal cancer models following FASN knockdown.^42^ These collective findings establish the dual role of FASN in suppressing apoptosis and promoting angiogenesis across multiple cancer types, thereby substantiating our hypothesis that FASN acts as the mechanistic executor through which the circHIPK3/ALYREF axis drives oncogenic effects in GBC. Our data confirmed FASN overexpression in GBC, aligning with previous reports of its oncogenic role in this malignancy.^43^ Moreover, the authors found that FASN overexpression functionally rescued the tumor-suppressive phenotypes induced by circHIPK3 knockdown in GBC cells. This epistatic relationship conclusively demonstrates that circHIPK3 exerts its pro-tumorigenic effects through the ALYREF-dependent regulation of FASN expression.

However, the present study has some limitations. First, this study acknowledges inherent limitations in its experimental models. The *in vitro* model employed inherently oversimplifies tumor microenvironments through the absence of human-specific stromal crosstalk, immune modulation, and physiological metabolic dynamics. Similarly, while the cell line-derived xenograft model provides valuable insights, it incompletely recapitulates the genetic diversity, natural disease evolution, and therapeutic response heterogeneity characteristic of human GBC. Additionally, interspecies divergence in xenobiotic metabolism, immunological pathways, and tumor-stroma signaling between murine models and humans may constrain the clinical translation of mechanistic findings. Future investigations will prioritize validation in spontaneous tumor models such as companion animals with naturally occurring malignancies, which better preserve species-specific microenvironmental interactions and therapeutic response profiles. Second, while the mechanistic focus on FASN was guided by its robust association with the circHIPK3/ALYREF axis in both bioinformatic predictions and experimental validation, the authors acknowledge that additional molecular players within circHIPK3′s broader ceRNA network or RBP interactome may cooperatively drive the observed oncogenic phenotypes. A critical next step will involve systematic delineation of circHIPK3’s network-level regulatory architecture in gallbladder carcinogenesis, including *in vivo* validation of its interaction with ALYREF and functional characterization of ALYREF’s role in tumor progression. Finally, the absence of comprehensive bioinformatic profiling constitutes another limitation of this work. To address this gap, the authors plan to implement circRNA sequencing initiatives coupled with multi-omics profiling strategies in follow-up studies to fully resolve the molecular landscape of circHIPK3-mediated oncogenesis.

## Conclusion

In summary, the present study reveals the critical role of circHIPK3 in regulating GBC. circHIPK3 promotes the expression and stability of FASN mRNA through inhibition of ALYREF ubiquitination, which promotes the proliferative, migratory invasive, and angiogenic capacity of GBC, and inhibits apoptosis. circHIPK3 is highly expressed in GBC patients and this high expression is associated with later TMN staging and lymph node metastasis. The present study provides new evidence and rationale for circHIPK3 as a diagnostic biomarker and therapeutic target for GBC.

## Availability of data and materials

The datasets used and/or analyzed during the present study are available from the corresponding author upon reasonable request.

## Ethics approval

The present study was approved by the Ethics Committee of Qinghai University Affiliated Hospital (n° 20181125QH23) and written informed consent was provided by all patients prior to the study start. All procedures were performed in accordance with the ethical standards of the Institutional Review Board and The Declaration of Helsinki, and its later amendments or comparable ethical standards.

The animal experiments were complied with the ARRIVE guidelines and performed in accordance with the National Institutes of Health Guide for the Care and Use of Laboratory Animals. The experiments were approved by the Institutional Animal Care and Use Committee of Qinghai University Affiliated Hospital (n° 20190618QH10).

## Authors’ contributions

ZhenWei Li designed the research study. ZhenWei Li, HaiJiu Wang and Cheng Wang performed the research. ZhiXin Wang and Zhi Xie provided help and advice. HaiJiu Wang, ZhiXin Wang and JinMing Liu analyzed the data. ZhenWei Li wrote the manuscript. Zhi Xie reviewed and edited the manuscript. All authors contributed to editorial changes in the manuscript. All authors read and approved the final manuscript.

## Funding

National Clinical Key Specialty Construction Project, Qing Health Office (2023) NO.125.

## Declaration of competing interest

The authors declare no conflicts of interest.

## Appendix Supplementary materials

Image, application 1
