# Supplementary material for: CircHIPK3′s dual role in promoting angiogenesis and inhibiting apoptosis through FASN mRNA stabilization in gallbladder cancer
Source: Clinics (Sao Paulo). 2025 Jun 3;80:100697. doi: 10.1016/j.clinsp.2025.100697 (PMC12171766; doi:10.1016/j.clinsp.2025.100697)

**CLINICS-D-25-00056_ Supplementary Materials**

**Supplementary Table 1** Ten candidate RNA Binding Proteins (RBPs) interacting with circHIPK3 identified in the ENCORI platform based on the highest ClipSiteNum score.

| **RBP** | **ClusterNum** | **ClipExpNum** | **ClipSiteNum** |
| --- | --- | --- | --- |
| TARDBP | 19 | 36 | 166 |
| ELAVL1 | 40 | 17 | 82 |
| HNRNPC | 23 | 11 | 47 |
| U2AF2 | 19 | 10 | 45 |
| HNRNPA2B1 | 18 | 4 | 33 |
| ALYREF | 9 | 8 | 24 |
| CTCF | 8 | 3 | 21 |
| IGF2BP1 | 9 | 9 | 20 |
| RNPS1 | 13 | 3 | 20 |
| RBMX | 15 | 6 | 19 |

**Supplementary Figure 1 Expression spectrum of circHIPK3 in cancer.** (A) Expression level of circHIPK3 in different types of cancer according to MiOncoCirc database. (B) Volcano plot of differentially expressed circRNAs in the GSE148561 dataset via the circMine platform, the adjusted p-value < 0.05 and the value of log-fold change |logFC| ≥ 1 were set as cutoff criteria.


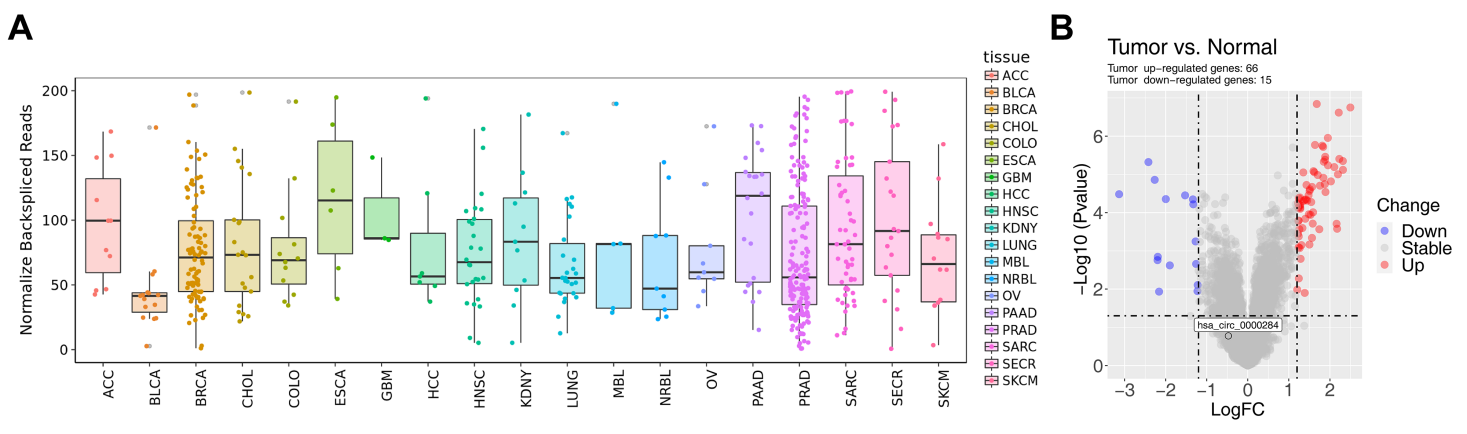


**Supplementary Figure 2** **Differential expression of ALYREF and its impact on cholangiocarcinoma patient survival according to GEPIA database.** (A) Expression level of ALYREF in cholangiocarcinoma according to GEPIA database. (B) Kaplan-Meier survival analysis the association between ALYREF expression level and survival of cholangiocarcinoma patients according to GEPIA database.


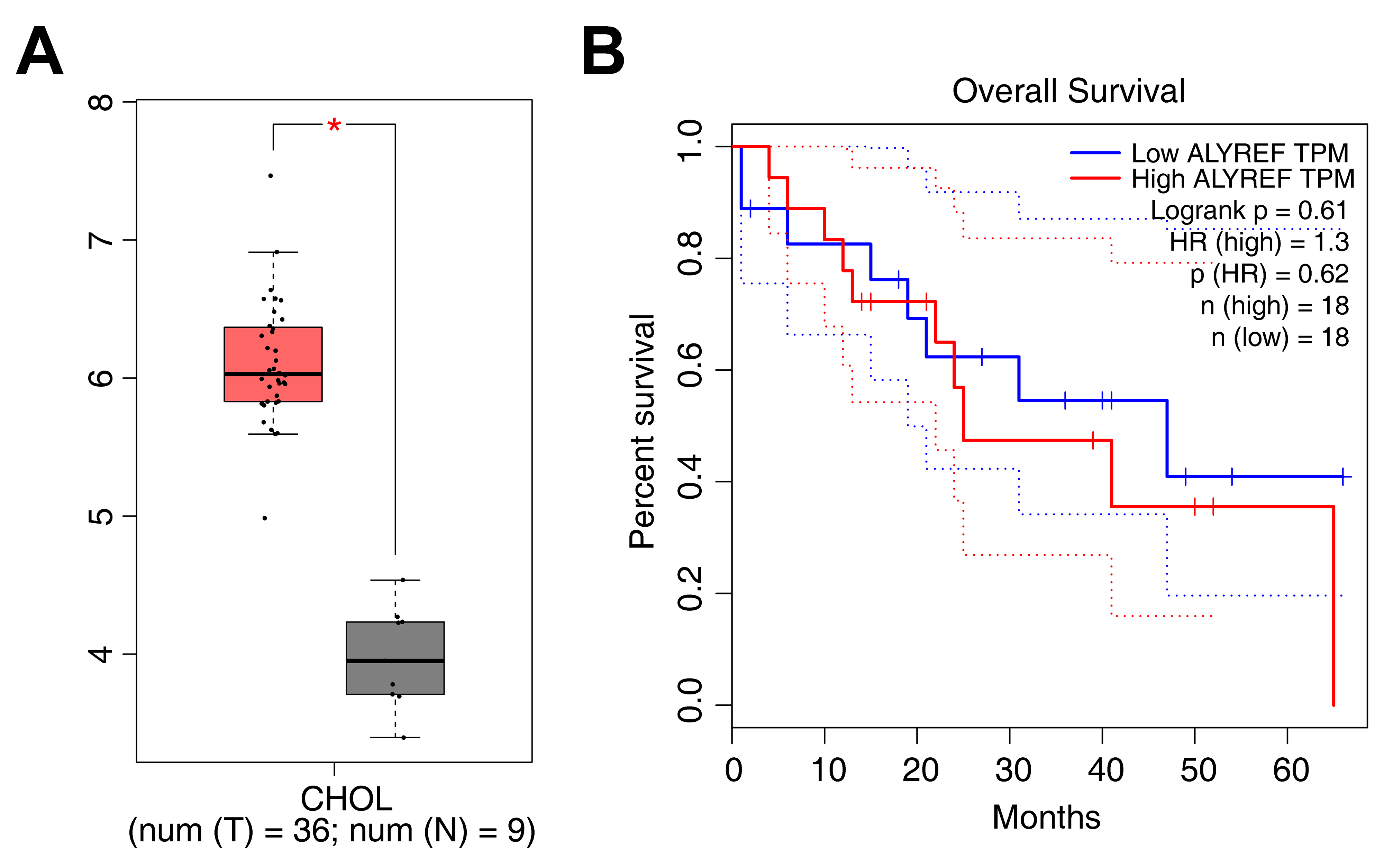

Supplement: Supplementary file 1 [file mmc1.docx]
